# Ontology-Based Querying with Bio2RDF’s Linked Open Data

**DOI:** 10.1186/2041-1480-4-S1-S1

**Published:** 2013-04-15

**Authors:** Alison Callahan, José Cruz-Toledo, Michel Dumontier

**Affiliations:** 1 Department of Biology, Carleton University, 1125 Colonel By Drive, Ottawa, ON, Canada; 2 Institute of Biochemistry, Carleton University, 1125 Colonel By Drive, Ottawa, ON, Canada; 3 School of Computer Science Carleton University, 1125 Colonel By Drive, Ottawa, ON, Canada

## Abstract

**Background:**

A key activity for life scientists in this post “-omics” age involves searching for and integrating biological data from a multitude of independent databases. However, our ability to find relevant data is hampered by non-standard web and database interfaces backed by an enormous variety of data formats. This heterogeneity presents an overwhelming barrier to the discovery and reuse of resources which have been developed at great public expense.To address this issue, the open-source Bio2RDF project promotes a simple convention to integrate diverse biological data using Semantic Web technologies. However, querying Bio2RDF remains difficult due to the lack of uniformity in the representation of Bio2RDF datasets.

**Results:**

We describe an update to Bio2RDF that includes tighter integration across 19 new and updated RDF datasets. All available open-source scripts were first consolidated to a single GitHub repository and then redeveloped using a common API that generates normalized IRIs using a centralized dataset registry. We then mapped dataset specific types and relations to the Semanticscience Integrated Ontology (SIO) and demonstrate simplified federated queries across multiple Bio2RDF endpoints.

**Conclusions:**

This coordinated release marks an important milestone for the Bio2RDF open source linked data framework. Principally, it improves the quality of linked data in the Bio2RDF network and makes it easier to access or recreate the linked data locally. We hope to continue improving the Bio2RDF network of linked data by identifying priority databases and increasing the vocabulary coverage to additional dataset vocabularies beyond SIO.

## Background

A key activity for life scientists in this post “-omics” age involves searching for and integrating biological data from the multitude of independent online biological databases. This task usually involves a tedious manual search and assimilation of isolated and diverse collections of life sciences data hosted by both large organizations such as the National Center for Biotechnology Information (NCBI) [1] and the European Bioinformatics Institute (EBI) [2], as well as smaller groups such as the one that publishes iRefIndex [3], a database of molecular interactions aggregated from 13 data sources. While some resources provide links to other databases (*e.g.* UniProt links its entries to hundreds of other databases [4]), these often lack a semantic richness required to understand the intent or limitation of the linkage. With thousands of biological databases and hundreds of thousands if not millions of datasets, our ability to find relevant data is hampered by non-standard interfaces backed by an enormous diversity of data formats [5]. Our inability to easily navigate through available data and databases presents an overwhelming barrier to their reuse.

The open-source Bio2RDF project [6-8] uses Semantic Web technologies and a set of conventions to provide linked data [9] for the life sciences. It consists of scripts that automatically download and convert well known biological data sets into the Resource Description Framework (RDF) from their original formats, whether it be flat-files, tab-delimited files, XML or SQL. Using the powerful SPARQL Protocol and RDF Query Language (SPARQL), Bio2RDF linked data can be uniformly explored and queried.

Although there are several efforts for provisioning life science linked data such as Neurocommons [10], LinkedLifeData [11], W3C HCLS [12], Chem2Bio2RDF [13] and BioLOD [14], Bio2RDF is unique in several ways. First, Bio2RDF attempts to capture the intended meaning serialized by the original data providers in both content and structure. Each Bio2RDF dataset has a unique linked data vocabulary and topology and does not attempt to marshal the data into a common schema. Second, Bio2RDF relies on a set of basic guidelines to produce syntactically interoperable linked data across all datasets. Third, Bio2RDF infrastructure provides a federated network of SPARQL endpoints and provisions the community with an expandable global network of mirrors that host Bio2RDF datasets. Finally, Bio2RDF is open source and freely available for to use, modify or redistribute.

Although Bio2RDF facilitates integration of and programmatic access to otherwise heterogeneous datasets (in both content and format), a complete syntactic and semantic normalization across the numerous datasets has yet to be fully realized. This is partially because, as stated above, each Bio2RDF dataset has a unique structure and vocabulary. Linked data serialized as RDF also inherently lacks complex formal semantics that would allow a reasoner to infer the relationship between data items in different datasets. As such, the *meaning* of types and relations in linked data records and between entities from different datasets are, at best, weakly defined. Consequently, there is no integration at the level of relations or types, and as a result Bio2RDF data cannot currently be queried with a universal data model.

Several approaches for integrating biological data have been reported [15-17], but they rely on a variety of data formats and standards, making sustainable large-scale integration challenging [18]. Ontologies have also been used as a means to integrate data at a global level [19-23] but these efforts typically involve creating a new application ontology or re-using domain specific ontologies. Here, we report the mapping of Bio2RDF dataset vocabularies to the Semanticscience Integrated Ontology (SIO) [24], an ontology that is being used to integrate SADI-based Semantic Web services [25-27]. At its core, SIO focuses on three kinds of basic entities: objects, processes and their attributes, from which over a thousand more specific kinds of entities are available. Importantly, SIO provides a coordinated set of relationships that can be used to richly and axiomatically describe entity types. Thus, if Bio2RDF types and relations were formally mapped to SIO, it would be possible to use SIO to query across and within Bio2RDF datasets.

Here, we describe a major update to the Bio2RDF project including consolidation and improvements to conversion scripts, new and updated datasets, and the ability to uniformly query Bio2RDF datasets using an integrated ontology for the sciences.

## Results

This section contains an overview of new and updated Bio2RDF datasets. We describe SPARQL 1.1 federated queries and SIO-based querying of Bio2RDF datasets.

### New and updated Bio2RDF linked data

Table 1 lists theBio2RDF datasets that are currently available at 19 SPARQL endpoints and for download. The following datasets are new to the Bio2RDF network:

**1. BioModels**– an EBI resource providing details on published computational models primarily from systems biology

**2. InterPro** – an EBI resource that describes predicted protein classifications, domains and biologically significant sites

**3. BioPortal**– A collection of over 300 bio-ontologies from multiple providers

**4. NDC –** The National Drug Code Directory is a Food and Drug Administration (FDA) resource providing a current list of all drugs produced or otherwise processed for distribution by drug companies

**5. SABIO-RK** – An expert-curated biochemical reactions kinetics database that includes information about reaction participants, conditions and kinetics

**Table 1:** Bio2RDF datasets currently available

Dataset	Namespace	# of triples	# of unique subjects	# of unique predicates	# of unique objects
**Affymetrix**	affymetrix	44469611	1370219	79	13097194

Biomodels*	biomodels	589753	87671	38	209005

**Comparative Toxicogenomics Database**	ctd	141845167	12840989	27	13347992

**DrugBank**	drugbank	1121468	172084	75	526976

**NCBI Gene**	ncbigene	394026267	12543449	60	121538103

**Gene Ontology Annotations**	goa	80028873	4710165	28	19924391

**HUGO Gene Nomenclature Committee**	hgnc	836060	37320	63	519628

**Homologene**	homologene	1281881	43605	17	1011783

InterPro*†	interpro	999031	23794	34	211346

**iProClass**	iproclass	211365460	11680053	29	97484111

**iRefIndex†**	irefindex	31042135	1933717	32	4276466

**Medical Subject Headings**	mesh	4172230	232573	60	1405919

National Center for Biomedical Ontology*†	ncbo	15384622	4425342	191	7668644

National Drug Code Directory*	ndc	17814216	301654	30	650650

**Online Mendelian Inheritance in Man**	omim	1848729	205821	61	1305149

**Pharmacogenomics Knowledge Base**	pharmgkb	37949275	5157921	43	10852303

SABIO-RK*	sabiork	2618288	393157	41	797554

**Saccharomyces Genome Database**	sgd	5551009	725694	62	1175694

**NCBI Taxonomy**	taxon	17814216	965020	33	2467675

**Total**	**19**	**1010758291**	**57850248**	**1003**	**298470583**

The Bio2RDF datasets currently available for SPARQL querying and download at http://bio2rdf.org. The total number of triples, number of unique subject, number of unique predicates and number of unique objects are listed along with the Bio2RDF namespace for each dataset.

* Datasets new to the Bio2RDF network

† InterPro contains 13 domain resources, iRefIndex contains 13 interaction resources, and NCBO contains 107 OBO ontologies.

**Figure 1:**
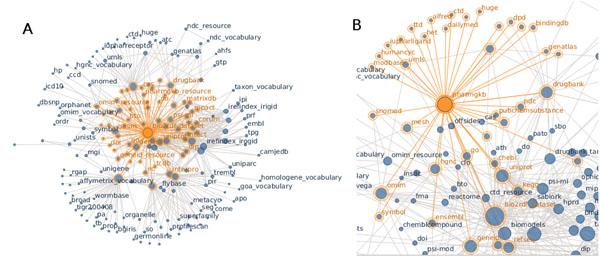
**Bio2RDF datasets showing namespace connectivity** Circles represent Bio2RDF datasets and the links between them represent a relation between one dataset and another based on IRI namespaces. Datasets with many links may be considered ‘hubs’ that serve to connect multiple datasets in the Bio2RDF network. **A** All Bio2RDF datasets and their namespace connectivity. **B** Detail of namespace connectivity for the PharmGKB dataset.

Of the 19 datasets, InterPro, BioPortal and iRefIndex are collections of multiple individual datasets. InterPro contains 12 datasets: CATH, Gene3D, PANTHER, PIRSF, Pfam, PRINTS, ProDom, PROSITE, HAMAP, SMART, SUPERFAMILY and TIGRFAMs. iRefIndex consists of 13 datasets (BIND, BioGRID, CORUM, DIP, HPRD, InnateDB, IntAct, MatrixDB, MINT, MPact, MPIDB, MPPI and OPHID). The Bio2RDF version of BioPortal currently only consists of 107 Open Biomedical Ontologies (OBO) ontologies including ChEBI, Protein Ontology and the Gene Ontology, and efforts are being made to coordinate with the NCBO BioPortal team to make use of their emerging SPARQL endpoint.Each dataset in the Bio2RDF network is connected to all the other datasets, either directly through a named reference or indirectly through some path through the data. Figure 1A shows how the new and updated Bio2RDF datasets are interconnected while Figure 1B shows detailed connectivity for the Pharmacogenomics Knowledge Base (PharmGKB). PharmGKB links to the following datasets (with their corresponding namespace): Allele Frequency Database (alfred), BindingDB (bindingdb), Chemical Entities of Biological Interest (chebi), Comparative Toxicogenomics Database (ctd), NLM’s DailyMed (dailymed), Health Canada Drug Product Database (dpd), DrugBank (drugbank), Ensembl (ensembl), GenAtlas (genatlas), NCBI Gene (geneid), PDB Heteroatom Vocabulary (het), Database of Human Unidentified Gene-Encoded Large Proteins Analyzed (huge), Human CYC (humancyc), International Union of Pharmacology Ligands (iupharligand), Kyoto Encyclopedia of Genes and Genomes (kegg), Medical Subject Headings (mesh), ModBase Database of Comparative Protein Structure Models (modbase), National Drug Code Directory (ndc), Online Mendelian Inheritance in Man (omim), PubChem (pubchem), Refseq (refseq), Systematized Nomenclature of Medicine (snomed), HGNC Gene Symbols (symbol),Therapeutic Targets Database (ttd), and Uniprot (uniprot). Several of these datasets (chebi, ctd, drugbank, geneid, kegg, mesh, ndc, omim, pubchem, refseq, snomed, symbol, uniprot) are part of the Bio2RDF network, and can be further explored by following the linked data or through federated queries.

Federated queries make it possible to formulate a query across connected datasets that reside in separate SPARQL endpoints. The following SPARQL query makes it possible to query SGD, Gene Ontology Annotations and the Gene Ontology to obtain the set of genes that encode proteins involved in zinc ion binding:

The first five results of the query are presented in Table 2. This query returns four variables for each result, defined by a SELECT statement: the gene IRI, the gene label, a description provided for the gene, and the protein IRI. These possible values of these variables are restricted by the conditions in the WHERE clause, which uses structure of the SGD linked dataset to retrieve the desired results. The query uses a SERVICE [28] clause to query the remote SPARQL endpoint for GO identifiers with the label “zinc ion binding”, and uses the resulting GO identifiers to restrict the top part of the query, which searches for SGD genes with corresponding GO annotations.

**Table 2:** Top 5 results of a federated SPARQL query to search for SGD genes related to the GO function with label “zinc ion binding”

Gene identifier	Gene label	Description	Protein identifier
**http://bio2rdf.org/sgd:S000004688**	YMR083W	Mitochondrial alcohol dehydrogenase isozyme III	http://bio2rdf.org/sgd:S000004688gp

**http://bio2rdf.org/sgd:S000000349**	YBR145W	Alcohol dehydrogenase isoenzyme V	http://bio2rdf.org/sgd:S000000349gp

**http://bio2rdf.org/sgd:S000002624**	YDR216W	Carbon source-responsive zinc-finger transcription factor	http://bio2rdf.org/sgd:S000002624gp

**http://bio2rdf.org/sgd:S000000819**	YER017C	Component of the mitochondrial inner membrane m-AAA protease	http://bio2rdf.org/sgd:S000000819gp

**http://bio2rdf.org/sgd:S000001306**	YIL044C	ADP-ribosylation factor (ARF) GTPase activating protein (GAP) effector	http://bio2rdf.org/sgd:S000001306gp

SGD gene identifier, label, partial description and corresponding protein identifier for each query result

### Bio2RDF vocabulary mappings to SIO

Table 3 lists the Bio2RDF datasets whose vocabularies (dataset-specific types and relations) have been manually mapped to SIO. A total of 136 classes and 407 object properties across all Bio2RDF datasets were mapped to SIO. Table 3 contains the number of classes and object properties in the corresponding dataset vocabulary ontology as well as the number of exact and intermediate subclass matches. Exact matches are those mappings for which a SIO class was found to be the most specific parent class for the corresponding Bio2RDF dataset vocabulary class. Intermediate matches are mappings for which a SIO class was determined to be a parent class for the dataset vocabulary but for which a more specific parent could not be identified in SIO.

**Table 3:** Bio2RDF vocabulary and SIO mapping metrics

Dataset	# of classes	# of object properties	# of class exact mappings	# of class intermediate mappings
**Affymetrix**	1	15	0	1

**BioModels**	0	2	0	0

**CTD**	4	3	3	1

**DrugBank**	15	58	6	9

**GO Annotations**	1	2	0	1

**HGNC**	1	30	0	1

**Homologene**	1	5	0	1

**InterPro**	7	21	2	5

**iRefIndex**	5	6	0	5

**MeSH**	3	46	0	3

**OMIM**	7	47	3	4

**NCBI Taxonomy**	4	16	1	3

**NCBI Gene**	18	89	1	17

**NDC**	11	16	0	11

**PharmGKB**	16	8	6	10

**SABIO-RK**	0	3	0	0

**SGD**	42	40	7	33

The number of classes and object properties mapped to SIO for each Bio2RDF dataset vocabulary ontology, as well as details on quality of class mappings. Exact matches indicate that the Bio2RDF vocabulary class is a direct subclass of its mapped parent SIO class. Intermediate matches are those that are not exact matches in the SIO class hierarchy but for which there was not a more precise parent class candidate.

### Querying Bio2RDF linked data using SIO

The availability of Bio2RDF-SIO mappings makes it possible to compose data source independent SPARQL queries that can be applied to all SPARQL endpoints, as opposed to *a priori* formulation of dataset specific queries against targeted endpoints.

**Figure 2:**
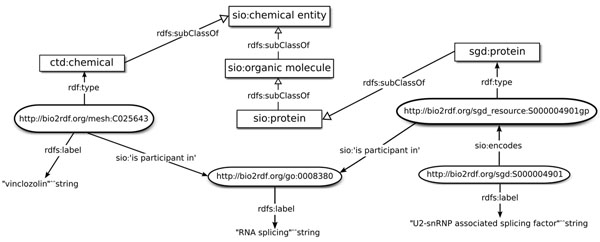
**Graphical representation of results of the SPARQL query for CTD chemicals that participate in the same molecular process as SGD proteins.** The SGD gene S000004901 encodes a gene product that is typed as ‘protein’ in the SGD dataset. This protein is annotated to be a participant in the GO process “RNA splicing”. Chemicals from the CTD dataset that are also participants in this GO process are retrieved. This query is possible because of cross-dataset links between CTD and SGD that result from mapping their Bio2RDF vocabularies to SIO: the SGD type ‘protein’ was mapped as a subclass of the SIO ‘protein’ class, while the CTD type ‘chemical’ was mapped as a subclass of the SIO ‘chemical entity’ class. The SGD dataset uses SIO relation ‘encodes’ to relate genes and gene products, and both the SGD and CTD datasets use the SIO relation ‘is participant in’ to link entities to GO processes. Square boxes are SIO OWL classes. Rounded boxes are RDF resources that are part of Bio2RDF linked datasets.

For instance, using the Comparative Toxicogenomics Database (CTD), SGD and the Gene Ontology, we ask for a chemical that participates in a process with an object that encodes a protein:

This query returns all bound variables specified in the WHERE clause, as indicated by the ‘SELECT *’ statement. This query again uses the SERVICE keyword to execute a federated query, in this case over the remote SGD SPARQL endpoint. In this query, the ‘a’ keyword is used as a short form for ‘rdf:type’. Among its answers, this query returns Vinclozolin (mesh:025643) and U2–snRNP associated splicing factor (sgd:S000004901), both of which are found to participate in RNA splicing (go:0008380) (Figure 2). This query is possible because the predicates ‘sgd_vocabulary:is-participant-in’ and ‘ctd_vocabulary:is-participant-in’ have both been mapped to the corresponding SIO object property ‘is participant in’, while ‘ctd_vocabulary:Chemical’ has been mapped to SIO class ‘chemical entity’ and ‘sgd_vocabulary:Protein’ has been mapped to SIO class ‘protein’.

## Discussion

While simple in structure, flat data files are charged with implicit semantics, especially in datasets where relationships between biological entities are taken for granted. Consider, for example, that the SGD entry for *gene* BCY1 (sgd:S000001295) provides functional annotations for its encoded gene products, primarily a *protein* product (which may not have a standard identifier). Encoding such semantics explicitly is a challenge when generating linked data from other data formats, but one which may have significant impact on how the data is subsequently incorporated for analysis. The consolidation, review and update of Bio2RDF conversion scripts created an opportunity to ensure that minimal local semantics were asserted. Where applicable, we disambiguated between dataset, record and entity level provenance, such that information that pertains to datasets (release versions) was recorded separately from record-level information (*e.g.* curators/editors, dates of creation, updates, *etc*.) and from entity-level information (biological or informational relationships).

Ontologies constructed using the Web Ontology Language (OWL) can be used to define the meaning of types and relations used in Linked Data. Although OWL formally differentiates classes, object properties (which relate two individuals), data properties (which have as value only literals) and individuals, the same cannot be said of all Bio2RDF datasets. In the case where a literal represents a label or a quantity, there is a need to transform it into an instance of a type (*e.g.* sio:label, sio:quantity, sio:description, *etc.*), nominally a SIO ‘information content entity’, with the literal attached to sio:has-value, which is SIO’s only datatype property. So while Bio2RDF datatype relations were mapped to SIO object properties, the challenge of rendering these literals using sio:has-value still remains. One solution would involve transforming SIO-containing queries to datatype mappings, and another is to transform all literal data into an instantiated class. In this way, it would become possible to categorize each kind of literal, as has been demonstrated with the Chemical Information Ontology [29].

Ontologies also enable data integration through querying using a unified vocabulary. By making use of SIO to map Bio2RDF vocabularies, not only can previously unconnected datasets be queried to discover the relationships between them, but the intended semantics of the classes and object properties they use are formally defined such that computational reasoning and inference is possible. Unlike upper level ontologies such as the Basic Formal Ontology (BFO) [30] and its associated Relation Ontology [31], SIO contains unified and rich axiomatic descriptions of its classes and properties. SIO also enables the representation of cumulative-constitutively organized material entities [32], which are common in biological domains but not captured by BFO. SIO also contrasts OBO domain ontologies such as the Vaccine Ontology [33] in that it does not suffer from a proliferation of classes and object properties as a result of attempting to integrate multiple OBO ontologies. Instead, SIO provides a well-described upper level that acts as an anchor for other domain specific classes and object properties as well as design patterns [34] for developing new classes and their axiomatic definitions. Finally, another advantage of mapping Bio2RDF vocabularies to SIO is that once new candidate classes are identified they can be added to SIO, thus improving the coverage of the ontology.

The process of manually mapping Bio2RDF dataset vocabulary classes to SIO identified branches of the SIO class hierarchy that can be further developed. Specifically, Table 3 indicates that many Bio2RDF dataset vocabulary classes do not have exact parent matches in SIO. For example, the Bio2RDF National Drug Code (NDC) vocabulary class ‘vaccine’ was mapped to SIO ‘heterogeneous substance’ (a subclass of ‘material entity’) as its direct parent. A search for the term ‘vaccine’ in NCBO BioPortal’s ontology index returns many hits, including a class in the Ontology for Biomedical Investigations (OBI) [35] and in SNOMED Clinical Terms [36]. Closer examination of the OBI ‘vaccine’ class reveals that its direct superclass is ‘material entity’, which is less specific than SIO’s ‘heterogeneous substance’. On the other hand, SNOMEDCT’s ‘vaccine’ class has the following class lineage:substance – biological substance – immunologic substance – immunologic agent – vaccine, immunoglobulin, and/or antiserum– vaccine. This indicates that the SNOMEDCT ‘vaccine’ class is more semantically granular than that of SIO. However, SIO ‘material entity’ has necessary (but not sufficient) axioms associated with it, specifically ‘has attribute *some* mass’ and ‘has proper part *only* material entity’, which are lacking in both OBI and SNOMEDCT.

Other Bio2RDF dataset vocabularies, such as OMIM, have exact superclass matches in SIO. For example, the OMIM vocabulary class ‘gene’ is mapped as a direct subclass of the SIO class ‘gene’ and OMIM class ‘entity’ is mapped as a direct subclass of SIO class ‘entity’. SIO ‘gene’ is a subclass of ‘nucleic acid part’, which is partially defined by the following axioms: ‘has direct part *some* nucleotide residue’ and ‘is part of *some* nucleic acid’. When a Bio2RDF vocabulary class has an exact parent match in SIO it becomes possible to take advantage of its rich hierarchy and axiomatic descriptions, but as shown above, in the case of less specific matches there may be better candidate parent classes in other bio-ontologies.

With access to more normalized structured data free of multi-valued fields, it becomes possible to consider converting Bio2RDFdatasets into full-fledged OWL ontologies. This has the significant advantage of allowing sophisticated reasoning to classify and check the consistency of the data itself, as was demonstrated in finding curation errors in the BioModels database [15]. Since there are only a handful of databases that contain raw experimental data (*e.g.* omics data such as that in PRIDE, a mass spectrometry database, or ArrayExpress, a microarray database) that *may* best remain as instance data, the rest could be fully formalized and gain the benefit of automated reasoning. Systematic conversion of Bio2RDF datasets to OWL may one day be possible, but will require significant effort and coordination to produce a unified knowledge base.

In the future we would like to facilitate integration of Bio2RDF data with other available bio-ontologies. The NCBO BioPortal team has mapped SIO to the domain ontologies they provide, based on lexical matching of class labels. This enables the querying of Bio2RDF resources using NCBO bio-ontologies, and provides an approach for integrating OBO ontologies with the Bio2RDF network via SIO. A related NCBO effort is its Resource Index [22], which uses the NCBO Annotator [37] to annotate text fields in records from 27 biological databases (including DrugBank, OMIM, PharmGKB, PubMed and UniProt) to link their content to NCBO bio-ontologies. We intend to contribute Bio2RDF to this index by executing the NCBO Annotator over Bio2RDF Linked Data. The resulting index would have the advantage that, unlike the databases currently included, NCBO ontologies would be linked to Bio2RDF data *and* the relations among datasets and ontologies would also be automatically available via the connectivity of Bio2RDF datasets.

Inter-namespace connectivity among datasets is useful for identifying how dataset content is related, but also for ranking candidate resources for addition to the Bio2RDF network. Specifically, if multiple Bio2RDF datasets contain relations to entities from a single biological database that is not currently in Bio2RDF, this database can be considered high priority for addition. Identifying candidate additions in this way is possible because of the Bio2RDF approach to consistent IRI creation as well as the resource registry which contains descriptions and preferred short names for datasets both in and not yet part of Bio2RDF.

Key to the continued growth of Bio2RDF is participation from the scientific community that produces and consumes the data that forms the Bio2RDF network. Housing the latest versions of all Bio2RDF data conversion scripts in GitHub allows others to both use Bio2RDF scripts to generate their own linked data graphs, but also to contribute new scripts that build on existing ones or convert datasets not currently in Bio2RDF. It should be noted that all Bio2RDF scripts are licensed using a free software license that permits re-use and modification with attribution. The GitHub repository may also act as a place for discussion in the community with regard to the Bio2RDF data modelling practices as well as additions to the project.

## Conclusions

Bio2RDF is an open source project to coordinate the provision of linked data for the life sciences. The use of an internally consistent IRI scheme across all datasets, mappings to other terminologies and publishing linked data at SPARQL endpoints facilitates the arduous task of data integration and knowledge discovery. Future work will focus on further integration of existing bio-ontologies with Bio2RDF datasets as well as prioritizing new databases to add to the Bio2RDF network.

## Methods

In this section we describe the tools and methods used to publish Bio2RDF data conversion scripts and linked data for the scientific community. We also present the Bio2RDF approach for modelling dataset-specific vocabularies with examples. Finally, we describe the mapping of Bio2RDF linked data vocabularies to SIO to enable querying across multiple datasets.

### Generating Bio2RDF linked data

A GitHub repository (http://github.com/bio2rdf/bio2rdf-scripts) was created to house Bio2RDF conversion scripts. We aggregated all known scripts (30 PHP scripts, 1 Java program and 1 Ruby gem) and updated them to address changes in the underlying data formats and content. GitHub users can download a copy of the repository, add or edit code and submit new scripts or changes, which will be reviewed by repository moderators.

Bio2RDF identifies data items using the following pattern:

http://bio2rdf.org/namespace:identifier

where the namespace is drawn from a resource registry of data providers and their preferred short name that currently maintained as part of PHP-LIB project [38]. For example, the Bio2RDF Internationalized Resource Identifier (IRI) for the UniProt entry with the identifier P26838 would be:

http://bio2rdf.org/uniprot:P26838

Two additional namespace patterns are used to specify new resources created in generating Bio2RDF data. The first is for dataset-specific types and predicates and follows the pattern:

http://bio2rdf.org/namespace_vocabulary:identifier

For example, the *Saccharomyces* Genome Database (SGD) describes genes and their protein products, which are typed as

http://bio2rdf.org/sgd_vocabulary:Protein

The second involves other resources created to convert n-ary relations into a set of binary relations, and follows the pattern:

http://bio2rdf.org/namespace_resource:identifier

For example, the Pharmacogenomics Knowledge Base (PharmGKB) describes associations between diseases, genes and drugs, but does not specify an identifier for these associations, and hence we assign a new stable identifier for each, such as

http://bio2rdf.org/pharmgkb_resource:association_PA445019_PA126

for the gene-disease association between cytochrome P450, family 2, subfamily C, polypeptide 9 (pharmgkb:PA126) and Myocardial Infarction (pharmgkb:PA445019).

Bio2RDF scripts were executed to generate linked data from the latest version of all available datasets (as of September 15, 2012).

### Mapping Bio2RDF types and predicates to SIO

To facilitate dataset-independent querying, types and predicates used in Bio2RDF datasets were manually mapped to the Semanticscience Integrated Ontology (SIO). Dataset types and predicates were declared as Web Ontology Language (OWL) classes or object properties as appropriate using the following SPARQL queries:

The resulting vocabularies were manually mapped to corresponding SIO classes and object properties. The mapping process involved asserting the rdfs:subClassOf relation for Bio2RDF vocabulary classes (*e.g. *sgd_vocabulary:Chemical is a subclass of sio:’chemical entity’), as well as owl:equivalentProperty and owl:superProperty relations as appropriate. All types used in Bio2RDF datasets were added in the corresponding vocabulary ontology and mapped to SIO. Only predicates belonging to a ‘[dataset]_vocabulary’ namespace, however, were mapped to SIO. RDF, RDFS, OWL and Dublin Core predicates were not included in the dataset vocabularies or mapped to SIO. Each Bio2RDF dataset vocabulary is serialized as an OWL ontology, and its mappings to SIO are serialized as a separate mapping ontology that imports the corresponding vocabulary ontology and SIO.

### Provisioning Bio2RDF datasets and SPARQL endpoints

Each Bio2RDF linked dataset was loaded into a unique SPARQL endpoint for querying, using OpenLink Virtuoso Community Edition build 06.01.3127 with the faceted browser, SPARQL 1.1 query federation, and Cross-Origin Resource Sharing (CORS) enabled. SPARQL endpoints are accessible at http://[namespace].bio2rdf.org. For example, the *Saccharomyces* Genome Database (SGD) SPARQL endpoint is available at http://sgd.bio2rdf.org. The list of all available endpoints can be found at http://bio2rdf.org. All updated Bio2RDF linked data is also available for download as N-Triples at http://download.bio2rdf.org. We have implemented a versioning and release protocol for Bio2RDF datasets and mappings. The current Release 2 of Bio2RDF data sets, featuring updates to 19 datasets, is available at http://download.bio2rdf.org/current/. The corresponding vocabulary-SIO mapping files are available at the GitHub bio2rdf-mapping project page, at http://github.com/bio2rdf/bio2rdf-mapping/tree/master/2/[namespace]* e.g. *https://github.com/bio2rdf/bio2rdf-mapping/tree/master/2/ctd.

## Competing interests

The authors have no competing interests to declare.

## Authors' contributions

AC, JCT and MD carried out updates to Bio2RDF data conversion scripts and migration to GitHub, as well as managed loading of datasets into Virtuoso. AC and JCT mapped Bio2RDF vocabularies to SIO, developed dataset queries, collected vocabulary metrics, and drafted the manuscript. MD conceived of the study, participated in its design and helped to draft the manuscript. All authors read and approved the final manuscript.

## Declarations

Publication in this supplement was supported by an Ontario Early Researcher Award to MD.

This article has been published as part of *Journal of Biomedical Semantics* Volume 4 Supplement 1, 2013: Proceedings of the Bio-Ontologies Special Interest Group 2012. The full contents of the supplement are available online at http://www.jbiomedsem.com/supplements/4/S1
